# Chondrosarcoma: A Clinical Review

**DOI:** 10.3390/jcm12072506

**Published:** 2023-03-26

**Authors:** Aaron Gazendam, Snezana Popovic, Naveen Parasu, Michelle Ghert

**Affiliations:** 1 Division of Orthopaedic Surgery, Department of Surgery, McMaster University, Hamilton, ON L8S 4L8, Canada; 2 Department of Pathology and Molecular Medicine, McMaster University, Hamilton, ON L8S 4L8, Canada; 3 Department of Radiology, McMaster University, Hamilton, ON L8S 4L8, Canada

**Keywords:** chondrosarcoma, review, sarcoma

## Abstract

Chondrosarcomas are a diverse group of malignant cartilaginous matrix-producing neoplasms. Conventional chondrosarcomas are a continuum of disease based on the biologic activity of the tumor. The tumors range from the relatively biologically benign low-grade tumors or intermediate atypical cartilaginous tumors (ACTs), to malignant, aggressive high-grade tumors. The clinical presentation, radiographic and pathologic findings, treatments and outcomes vary significantly based on the histologic grade of the tumor. Chondrosarcomas present a diagnostic dilemma, particularly in the differentiation between high- and intermediate-grade tumors and that of low-grade tumors from benign enchondromas. A multidisciplinary team at a tertiary sarcoma centre allows for optimal care of these patients.

## 1. Introduction

Chondrosarcomas are a rare malignant bone tumor arising from cartilage-producing cells. The present review will provide a comprehensive overview of the epidemiology, pathology, diagnosis, and treatment of chondrosarcoma. This review will also highlight emerging treatment modalities and promising areas for future research.

### Epidemiology

Chondrosarcomas are considered the second most common sarcoma of bone following osteosarcoma [1]. Chondrosarcoma accounts for 20–30% of all skeletal sarcomas and have an estimated incidence of 1 in 200,000 per year in the United States [2]. Recent literature has suggested that rates of chondrosarcoma are increasing and chondrosarcoma is now considered the most common primary bone malignancy in several countries due to the increase in ACTs diagnosed incidentally [3,4]. Chondrosarcoma has a mean age of presentation of 51, with over 70% of patients over the age of 40 at the time of diagnosis [2,5]. Notably, a rare subtype, mesenchymal chondrosarcoma, presents at a much younger age, with a peak incidence in the second and third decades of life. Chondrosarcoma demonstrates a slight predilection towards males; however, this varies by subtype [2,5].

Conventional primary chondrosarcomas are the most common variant and make up 85% of all cases [2]. Other rarer subtypes include secondary chondrosarcomas arising from benign precursors, and dedifferentiated, periosteal, mesenchymal and clear cell variants.

## 2. Clinical Presentation

### 2.1. Location

Chondrosarcomas most commonly present in the bony skeleton, although a small percentage present as a primary soft tissue mass [5]. These tumors can occur anywhere throughout the body, with the majority arising within the extremities (45%) followed by the axial skeleton (31%) [5]. Within the appendicular skeleton, there is a strong predilection for the long bones of the lower extremity, with the most common site of disease being the proximal femur. Within the long bones, the tumors generally originate in the medullary cavity of the metaphysis, with the exception of clear cell chondrosarcoma which originates in the epiphysis [6]. Chondrosarcomas also commonly originate in the pelvis, with approximately 20% of tumors originating from the pelvic bones [5]. Notably, periosteal chondrosarcomas and secondary peripheral chondrosarcomas arise from the surface of long bones with periosteal chondrosarcomas having a tendency for the distal femur. Mesenchymal chondrosarcoma demonstrates a more widespread distribution with involvement of the craniofacial bones, jaw, pelvis and vertebrae [7].

### 2.2. Signs and Symptoms

Similar to other primary bone malignancies, chondrosarcoma tends to present as progressive, insidious bony or joint pain that is worse at night [8]. Chondrosarcomas are often slow growing in nature and the duration of symptoms before diagnosis on average ranges from 10 to 15 months [9]. Some patients may present following a pathologic fracture due to the tumor invasion of the surrounding bone and weakening of the normal bony architecture, particularly in high-grade tumors [10]. In the case of periosteal chondrosarcoma or secondary chondrosarcoma arising from an osteochondroma, a palpable mass may be the first clinical sign and symptom [11]. Benign enchondromas are extremely common and can present in the setting of pain due to other causes such as rotator cuff tendinopathy. In these cases, correlation with imaging is critical.

Prompt diagnosis and treatment has the potential to reduce the disease burden and may help reduce the risk of metastatic spread and thus improve survival. At present, there are no novel detection methods that have been proven to improve early diagnosis. However, circulating tumor DNA has emerged as a potential promising biomarker to aid in both the diagnosis and residual disease detection in a range of tumors. In chondrosarcoma, isocitrate dehydrogenase (IDH) mutations are commonly found, and early studies suggest serum IDH DNA can be detected in patients with chondrosarcoma [12]. More research is needed to validate potential biomarkers and to determine their clinical utility in detecting chondrosarcoma at an early stage.

## 3. Imaging

### 3.1. Plain Radiographs

**Figure 1:**
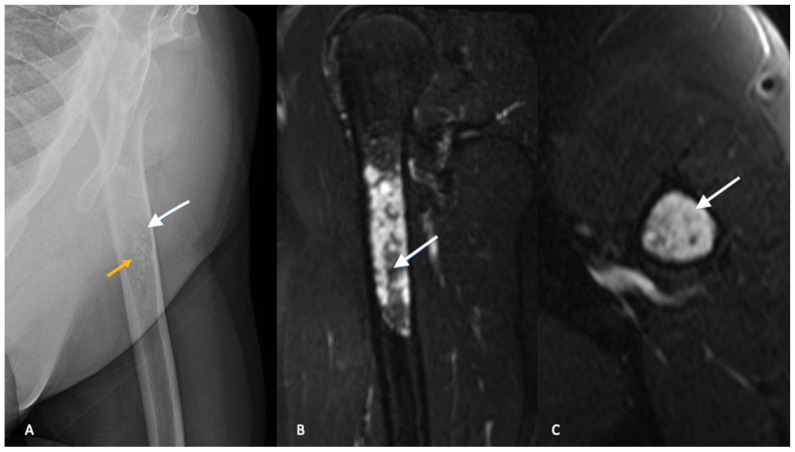
Radiograph (**A**) of the left proximal humerus demonstrates subtle lucency with internal stippled calcified foci (yellow arrow), in keeping with chondroid matrix. Subtle endosteal scalloping is present (white arrows) without cortical breakthrough or periosteal reaction. Sagittal (**B**) and axial (**C**) T2-weighted images with fat saturation of the same patient demonstrating a lobulated T2 hyperintense mass. A few stippled areas of low signal intensity represent chondroid matrix (white arrow). The lesion measures greater than 5 cm in length. Axial images demonstrate endosteal scalloping at the anterolateral border. No periostitis or cortical breakthrough. Imaging features are in keeping with an atypical cartilaginous tumor, which was confirmed on pathology after surgical curettage.

Plain radiographs with orthogonal views of the entire involved bone should be ordered as part of the initial workup. In conventional intramedullary chondrosarcoma, radiographs typically demonstrate mixed lesions with lytic and blastic activity [13]. Classically, the calcification pattern is described as “rings and arcs”, with the rings representing sclerosis and the arcs representing chondroid matrix. The distinction between ACTs and enchondroma is difficult and plain radiographs are not reliable in differentiating between the two entities [14,15]. Both ACTs and enchondromas tend to demonstrate a geographic lesion with lobular margins. Larger lesions (>5 cm) in the proximal metaphysis and endosteal scalloping favor a diagnosis of ACT [13,14] (Figure 1).

**Figure 2:**
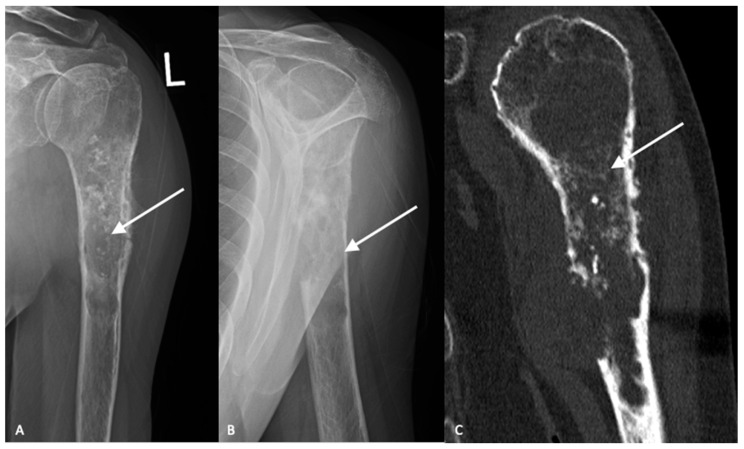
AP (**A**) and lateral (**B**) radiographs demonstrate an aggressive, heterogenous lytic lesion in the left proximal humerus with a wide zone of transition (white arrows). The lesion measures greater than 5 cm in length. Chondroid matrix is demonstrated. Periosteal reaction is present at the lateral aspect. There is a large area of cortical breakthrough at the anterior cortex, in addition to numerous areas of endosteal scalloping. Computed tomography (**C**) further demonstrates the cortical disruption and chondroid matrix formation (white arrow).

High-grade tumors usually present as larger lesions with less mineralization, with a moth-eaten appearance and permeative bone destruction (Figure 2A,B) [15]. Higher-grade lesions can cause more extensive endosteal scalloping that can result in cortical destruction or pathologic fracture. Soft tissue masses are seen in approximately 50% of cases. However, despite these characteristic differences, plain radiographs are not reliable in determining tumor grade [15].

### 3.2. Cross-Sectional Imaging

Magnetic resonance imaging (MRI) with and without contrast is the gold standard in diagnostic imaging for chondrosarcomas [16,17]. Magnetic resonance imaging of the entire bone should be performed to assess for skip lesions. MRI clearly demonstrates the extent of the tumor, invasion into the surrounding soft tissues and relationship to surrounding critical structures. Typically, chondrosarcomas demonstrate low intensity on T1-weighted images, high intensity on T2-weighted imaging with post-contrast enhancement depending on the histologic grade [18]. Computed tomography is the best modality to visualize bony destruction and the pattern of matrix mineralization but is not required for diagnosis (Figure 2C).

Similar to plain radiographs, differentiating between benign enchondromas and low-grade chondrosarcomas or ACTs is challenging with MRI. The findings of cortical thickening, intramedullary edema, bony expansion and entrapped fat are suggestive of an ACT but not diagnostic (Figure 1B,C) [19,20]. There continues to be significant interobserver variability and misdiagnosis with MRI as a diagnostic tool in the evaluation of ACTs and the diagnosis cannot be made on imaging alone [19].

**Figure 3:**
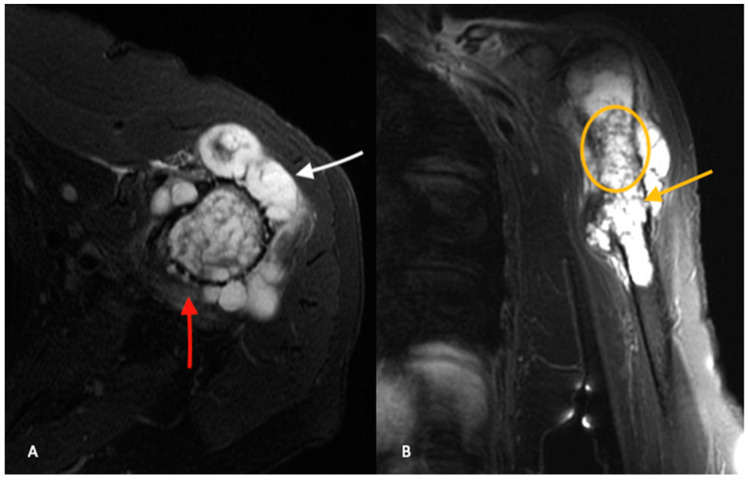
Axial (**A**) and coronal (**B**) T2-weighted, fat-saturated MR images of the same patient in Figure 2 confirm present of a chondroid lesion (yellow circle) with aggressive features, including cortical breakthrough (white arrow) and numerous areas of endosteal scalloping (yellow arrow). There is a significant amount of extra-osseous chondroid tumor. Perilesional edema (red arrow) is seen extending into the adjacent soft tissues.

When compared to ACTs, high-grade chondrosarcomas have a higher prevalence of loss of entrapped fatty marrow, cortical breakthrough and extraosseous soft tissue masses (Figure 3A,B) [18]. These findings are neither pathognomonic nor universally present and MRI findings must be considered in the setting of clinical and histologic findings when differentiating between ACTs and high-grade chondrosarcomas.

Secondary chondrosarcomas arising from osteochondromas represent a variant with unique imaging features. The section of the osteochondroma that dedifferentiates into chondrosarcoma is the cartilaginous cap. A cartilage cap thickness ≥2 cm is suggestive of the presence of a secondary chondrosarcoma [11,21]. However, there is a wide range of reported cartilage cap thickness in both osteochondromas and secondary chondrosarcomas and the size of the cap cannot be used in isolation to differentiate between the two [11].

## 4. Diagnosis and Staging

### 4.1. Biopsy

Biopsy and subsequent histologic grading of lesional tissue is imperative and helps to direct therapeutic decision making. However, histologic grading of cartilaginous tumors is challenging and is subject to high rates of interobserver variability [15,22]. Additionally, there is a high rate of biopsy sampling error, particular in pelvic-based lesions [23]. The preoperative histologic grade should be taken into consideration alongside radiologic and clinical assessment in determining optimal management [24].

Lesional tissue can be obtained through open surgical techniques or percutaneously, often with image guidance. Although open biopsy remains the gold standard, image guided percutaneous techniques offer several advantages including reduced cost, lower rates of tumor seeding and the ability to more readily biopsy deep lesions [25,26,27]. Core needle biopsies have demonstrated relatively high concordance with the final pathologic analysis in chondrosarcoma, particularly in long bone tumors [28,29].

### 4.2. Staging

Current guidelines recommend plain radiographs and cross sectional imaging of the entire involved bone to evaluate the lesion and assess for skip metastases [16,17]. Magnetic resonance imaging demonstrates the extent of tumor invasion into the surrounding tissues, relationship to critical structures and presence of skip lesions. In the setting of contradictions to MRI, computed tomography (CT) can be utilized. A CT scan of the chest should be performed in all patients to detect the presence of pulmonary metastases. Radionuclide bone scan with technetium-99 or whole body fluorodeoxyglucose positron-emission tomograph/CT (FDG PET/CT) are currently recommended to detect skeletal metastases [16,17].

There is no chondrosarcoma-specific staging system; therefore, chondrosarcomas are generally staged using either the Enneking classification or the American Joint Committee on Cancer (AJCC) Staging systems for bone sarcomas [30,31]. The Enneking classification is based on tumor grade, soft-tissue extension, and the presence of metastases. The AJCC also evaluates tumor size and location as they have been shown to have prognostic value in bone sarcomas.

## 5. Subtypes, Diagnostic and Molecular Pathology

### 5.1. Conventional Central Chondrosarcoma

Conventional central chondrosarcoma is the most prevalent variant and represents 85% of all chondrosarcomas with a peak incidence in the 5th to 7th decades of life [2]. These tumors have a predilection to the long bones and pelvis, with the most common sites of disease being the proximal and distal femur, proximal humerus, and pelvis [32]. As with other primary bone malignancies, disease presentation in the long bones confers a survival benefit when compared to chondrosarcoma in the pelvis and axial skeleton [33].

**Figure 4:**
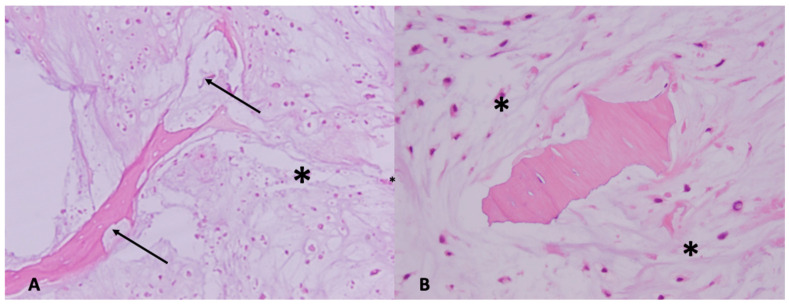
Microscopic images showing cartilaginous matrix-producing tumor (*) with permeation into the bone (1(**A**) H&E ×100; arrow) and entrapping the pre-existing lamellar bone (1(**B**) H&E ×200).

**Figure 5:**
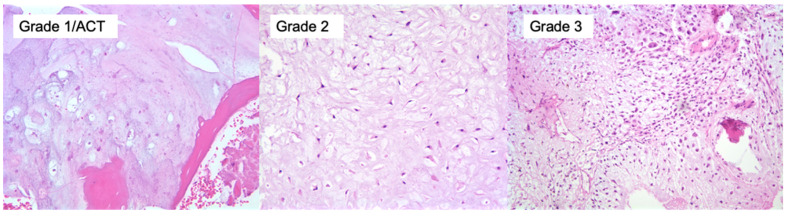
Microscopic images. Chondrosarcoma grading is based on the cellularity, cellular and nuclear pleomorphism/atypia and mitotic activity (H&E ×200). Grade 1/ACT is characterized by mild cellular atypia with small chondrocytes and nuclei. Grade 2 tumors are marked by increasing cellular atypia, increased nuclear size and binucleated cells. Grade 3 tumors are marked cellular atypia with nuclear enlargement and polymorphism.

On gross pathology, chondrosarcomas demonstrate a translucent lobular white cut surface which represents the hyaline cartilage [34]. Areas of mineralization present as yellow-white, chalky areas of calcium deposits. There may be cortical scalloping or destruction with associated soft tissue masses depending on tumor grade. Chondrosarcomas are graded on a scale of 1–3 according to the World Health Organization (WHO) [34]. The universal histopathological features of chondrosarcoma are the presence of hyaline cartilage matrix, architectural changes such as a permeative pattern, entrapment of the pre-existing lamellar bone trabeculae, myxoid matrix changes and cellular changes including increased cellularity, cellular atypia of chondrocytes, binucleated cells and variable mitotic activity (Figure 4). Tumor grade is based on cellularity, cellular/ nuclear atypia and mitoses (Figure 5). Importantly, grade 1 tumors of the appendicular skeleton (long and short tubular bones) are termed atypical cartilaginous tumors (ACTs) given their lack of overtly malignant behaviour. The term ‘grade 1 chondrosarcoma’ is used for low-grade axial cartilaginous tumors (flat bones—pelvis, scapula and skull base) as these tumors have poorer outcomes when compared to ACTs of the extremities [34].

There are overlapping histological features of enchondromas and central ACT/chondrosarcoma grade 1 (CS1). ACT/CS1 tumors demonstrate higher cellularity, irregular distribution of cells and more binucleated cells. More importantly, architectural criterion should be met including the presence of an entrapment growth pattern and an absence of encasement. Additionally, the presence of more than 20% myxoid matrix changes would favor the diagnosis of ACT/CS1 over enchondroma. Grade 2 tumors have increased cellularity, more prominent cellular and nuclear atypia and myxoid change compared to ACT/CS1. Grade 3 tumors demonstrate further increased cellularity, nuclear and cellular pleomorphism and easily found mitoses [34].

### 5.2. Secondary Chondrosarcomas

Secondary chondrosarcomas can arise centrally from a pre-existing enchondroma or peripherally from an osteochondroma. The risk of malignant transformation of an osteochondroma to a secondary chondrosarcoma is approximately 1% for solitary lesions and 5% for multiple lesions, although this is likely a gross overestimate due to the unknown number of humans with undetected osteochondromas [35,36]. In patients with Ollier’s Disease or Maffucci Syndrome, the risk of malignant transformation of an enchondroma is markedly elevated at 10–40% [11].

Grossly, secondary central chondrosarcomas appear similar to the conventional subtype, whereas peripheral secondary chondrosarcomas demonstrate a thickened cartilage cap (>2 cm) which may have cystic changes in the cartilaginous portion [34]. Secondary chondrosarcomas are most commonly low-grade tumors and the histological grading is similar to that of conventional tumors [11]. However, when secondary central chondrosarcomas arise from Ollier’s Disease or Maffucci Syndrome, it is difficult to distinguish between enchondromas and ACTs based on histopathological features such as cellularity and nuclear atypia. The differentiation is made based on a more infiltrative growth pattern alongside changes in clinical status and radiographs [11].

### 5.3. Rare Subtypes

The remaining 10–15% of chondrosarcomas are relatively rare subtypes with distinct clinical presentations, histopathology and radiographic findings that distinguish them from conventional chondrosarcoma (Table 1).

## 6. Molecular Characteristics

Given the resistance to systemic chemotherapy and radiotherapy, an understanding of the molecular characteristics and potential therapeutic targets is an area of interest. The Indian Hedgehog (IHH) pathway plays an important role in chondrocyte differentiation and upregulation of this pathway appears to play a role in the pathogenesis of conventional chondrosarcoma [40]. A number of targeted therapies have been studied and have demonstrated positive effects in animal models [41]. However, these therapies have failed to demonstrate significant benefit in phase 2 clinical trials [42,43].

Isocitrate dehydrogenase (IDH) represents a family of enzymes that play a role in the Krebs Cycle. Interestingly, mutations in the IDH1 and IDH2 genes are found in 50–70% of chondrosarcomas and are implicated in chondrosarcoma tumorigenesis [44]. IDH inhibitors have been investigated in a number of solid tumors, including chondrosarcoma [45,46]. However, IDH inhibitors have a significant toxicity profile and clinical data in chondrosarcoma are still limited. A number of trials are currently underway to determine the efficacy of IDH inhibitors on chondrosarcoma and other solid tumors [47].

The mTOR pathway and the PI3K-AKT signaling network are crucial regulators of cell metabolism, survival, and proliferation. Preclinical studies have shown the clinical relevance of these pathways in chondrosarcoma [48]. Small in vivo experiments have demonstrated mixed results with larger-scale trials needed [49]. Finally, the SRC pathway has also been proposed as a potential therapeutic target for the systemic management of chondrosarcoma. SCR proteins are cellular tyrosine kinases and tyrosine kinase inhibitors have been investigated in doxyrubicin-resistant chondrosarcoma, with some success in preclinical models [50].

## 7. Management

Each patient diagnosed with chondrosarcoma will require a tailored treatment plan. Factors including tumor location, grade and relationship to critical structures, alongside patient factors and the presence of metastases, must be evaluated by a multidisciplinary team to determine the optimal treatment plan for each patient.

Surgical management remains the mainstay of treatment for conventional chondrosarcoma as both radiation and chemotherapy have been shown to be ineffective. The relatively slow growth and low mitotic division and restricted drug penetration due to the poor vascularity of the tumor makes it resistant to conventional chemotherapy and radiation therapy [51].

With the exception of ACTs, the majority of chondrosarcomas are treated with wide surgical excision. Historically, these patients were treated with amputation to maximize local control and reduce the risk of metastases. However, advances in cross-sectional imaging and reconstruction options, the majority of patients are able to undergo limb-salvage procedures [52]. Modern limb-salvage techniques allow for similar survival rates and improved functional outcomes when compared to amputations [53]. Given this, primary amputation for the management of extremity chondrosarcoma is usually reserved for patients with very extensive and invasive disease [54].

Obtaining negative surgical margins is paramount as this may be the only modifiable risk factor in the treatment of chondrosarcoma [55,56]. However, what constitutes an adequate negative margin varies significantly in the literature and has not been accurately defined [55,57]. In intermediate- and high-grade chondrosarcomas, positive surgical margins are significant risk factors for both local recurrence and disease-specific survival [55,56,57].

Reconstruction options vary widely and depend on patient characteristics, tumor factors and surgeon preference. The choice to reconstruct must take into account patient considerations including age, functional demands and expectation of the patient. Patients who are older or with significant comorbidities may be too high risk for a complex and extensive reconstruction operation [54,58]. The tumor location, size and relationship to important structures including joints and neurovascular structures also dictate the resection and subsequent reconstruction. Endoprosthetic reconstructions have increased in popularity and tend to be the most common modern reconstruction options in patients with chondrosarcoma [59,60]. Other options involve allograft or allograft/prosthetic composite (APC) reconstructions. Given the advances in endoprosthetic reconstructions and the older age of presentation in chondrosarcoma, they are the mainstay for reconstruction following tumor resection.

### 7.1. Intrapelvic Tumors

Pelvic chondrosarcomas represent a challenging subset of patients with generally poor survivorship when compared to chondrosarcomas of the extremities [61]. Patients with pelvic tumors tend to present later, with larger tumors. Given the complex anatomy of the pelvis and proximity to critical structures, resection of pelvic chondrosarcomas is challenging and has high rates of positive margins and complications [62].

Importantly, grade 1 pelvic chondrosarcomas should not be called ACTs by the WHO given their higher rates of misdiagnosis, recurrence and metastatic potential [34]. Given this, wide resection with negative margins is recommended for pelvic chondrosarcomas of any grade [62]. Surgical management of pelvic-based chondrosarcomas consists of either a limb-salvage internal hemipelvectomy or hindquarter amputation. When deciding between limb salvage and amputation, the ability to conserve the sciatic nerve, femoral neurovascular bundle and the hip joint must be considered. Generally, preservation of two of these vital structures is required for limb salvage.

If a limb-salvage procedure is undertaken, the need for and type of reconstruction are individualized based on tumor and patient factors. Lesions isolated to the iliac wing (Type I) or pubic rami (Type III) can often be treated with resection alone. Type II resections of the periacetabulum represent the most challenging operations in both resection and reconstruction. One option is the Friedman–Eilber resection in which the tumor is resected and the pelvis is left unreconstructed [63]. Although this requires a long recovery time and may reduce functional outcomes, it avoids many of the complications that are associated with reconstruction. There are a variety of reconstruction options including endoprosthetic prosthesis or allograft/prosthetic composites. The ice-cream prosthesis is a modern option that is fixed into the ilium and has demonstrated improved functional outcomes and reduced complications compared to historical methods [64,65].

There is a growing body of literature that advocates for the use of computer navigation in the resection of pelvic tumors. Intraoperative computer navigation is thought to allow for improved intraoperative accuracy of tumor resections, allowing for negative margins while minimizing excessive healthy tissue resection [66,67]. However, the current data are limited to short-term case series and larger, prospective studies are required to validate its use.

In patients with extensive disease in which a resection would not provide clear margins, hindquarter amputation can be considered. With modern anesthetic and perioperative techniques, hindquarter amputations carry a 1% perioperative mortality and require prolonged recovery [68]. Recent literature has demonstrated that younger patients have improved overall survival with reasonable functional outcomes [69].

### 7.2. Atypical Cartilaginous Tumors

ACTs are low-grade locally aggressive tumors of the appendicular skeleton and are considered as intermediate tumors by the 2020 WHO classification due to their limited metastatic potential [34]. The current standard of care for the treatment of ACTs is intralesional curettage, with the consideration of adjuvants including phenol, ethanol or cryotherapy [70]. Cement or bone grafting is utilized to fill the defect with prophylactic surgical stabilization if required. Current literature suggests that intralesional curettage yields similar recurrence and metastatic rates when compared to more wide resection procedures [71]. However, in the cases of recurrent ACTs, wide excision is recommended as recurrence is suggestive of aggressive biologic behaviour [72]. Although secondary chondrosarcomas are primarily low-grade tumors, they represent a distinct entity and should undergo wide resection as intralesional curettage has demonstrated high recurrence rates [11,21].

## 8. Metastatic Disease

While chondrosarcoma is a slow-growing tumor, it has the potential to metastasize to other bones and soft tissues, with the lung being the most common site of metastasis. The risk of metastasis varies depending on the grade of the tumor, with high-grade and de-differentiated tumors having the highest risk of metastases.

The mechanism of metastasis in chondrosarcoma is not fully understood but it is believed to involve several factors, including the size and location of the primary tumor, the presence of dedifferentiated components, and the invasiveness of the tumor. Chondrosarcomas can also secrete various factors that promote tumor growth and metastasis, including matrix metalloproteinases and vascular endothelial growth factor. The treatment of metastatic chondrosarcoma typically involves systemic therapy, with surgical resection of primary bony tumor being advocated for by some authors [73].

### Systemic Therapy and Advanced Disease

For patients with advanced chondrosarcoma due to unresectable tumors or metastatic spread, treatment options are limited due to their poor response to both chemotherapy and radiation. Given the lack of efficacy, there are no standard recommendations for chemotherapy regimes for patients with advanced conventional chondrosarcoma. There are some retrospective data that support the use of doxorubicin and cisplatin for modest improvements in overall survival; however, toxicities must be considered in light of the minimal efficacy of these drugs in the clinical setting of chondrosarcoma [74,75].

Mesenchymal chondrosarcomas are a rare variant that occur in younger patients and have distinct clinical features. Several retrospective studies have demonstrated reduced recurrence and increased survival with (neo)adjuvant chemotherapy [76,77]. Current guidelines from both the National Comprehensive Cancer Network (NCCN) and the European Society of Medical Oncology (ESMO) advocate for chemotherapy regimens similar to those utilized in patients with Ewing’s Sarcoma [16,17].

Dedifferentiated chondrosarcoma is another variant that should be considered separately with respect to systematic therapy. Dedifferentiated chondrosarcomas are extremely biologically aggressive, with poor survival rates. Chemotherapy has demonstrated improved disease-free survival in this variant and current guidelines suggest that patients with dedifferentiated chondrosarcoma could be considered candidates for osteosarcoma regimes [16,74,78]. However, these regimens often cannot be tolerated in the population in which these tumors present (older adults and elderly). Dedifferentiated chondrosarcomas often express PD-L1 and there are ongoing trials evaluating the efficacy of biologic agents targeting PD-L1 (NCT04458922) [79].

## 9. Prognosis

The prognosis of chondrosarcoma varies widely and is based on tumor grade, stage and subtype. Atypical cartilaginous tumors have excellent survival with 5-year survival rates >90% [4]. Similarly, secondary chondrosarcomas have high 5-year survival rates of approximately 90% [11,21,80,81]. Survival worsens with increasing tumor grade, and 5-year survival rates for grade II and grade III chondrosarcomas are 75% and 30%, respectively [4]. The presence of metastases at presentation is an independent predictor of survival. Patients with metastatic disease at presentation have a 5-year survival rate of 28%, with a median overall survival of 14 months in a recent database study [82]. Other negative prognostic factors include increasing age, tumor size and tumors located in the axial skeleton [4].

### Subtypes

Dedifferentiated chondrosarcoma represents a particularly aggressive subtype, with a poor 5-year survival rate of between 0% and 24% [2,83]. Based on registry data, the 5-year survival rate of mesenchymal chondrosarcoma is 50% and worsens with increasing age and axial-based tumors [84]. Clear cell and juxtacortical subtypes most commonly present as lower-grade tumors and have 5-year survival rates of between 62% and 100% and 68% and 93%, respectively [2,37,85].

## 10. Conclusions

Chondrosarcomas are a heterogenous group of cartilage forming neoplasms and represent the second most common primary bone malignancy. The diagnosis and grading of chondrosarcoma remain challenging, in particular on a biopsy specimen, and treatment decisions should be made by a multidisciplinary team. Surgical management remains the mainstay of treatment as chondrosarcoma is resistant to both radiation and chemotherapy. Novel targeted therapies have shown promise in preclinical studies but future trials are needed to determine their efficacy in the clinical setting. Survival rates vary significantly based on tumor grade and presence of metastases.

## Figures and Tables

**Table 1:** Characteristics of rare chondrosarcoma variants [6,7,34,37,38,39].

SUBTYPE	Age	Location	Radiology	Histopathology	
Dedifferentiated	60–80	Femur, pelvis, humerus	Aggressive, destructive cartilaginous tumor	Low-grade chondrosarcoma with abrupt transition to high-grade non-cartilaginous sarcoma	+ PD-L1 + IDH1
Periosteal	20–40	Metaphyseal of long bones (femur and humerus)	Large lesions (>5 cm), arise from periosteum. Rarely medullary canal involvement	Resemble grade I–II conventional chondrosarcoma	+ IDH1
Mesenchymal	20–30	Wide variation, including extraskeletal soft tissue involvement	Primarily lytic, aggressive with wide zone of transition	Biphasic; portions of poorly differentiated small round or spindled mesenchymal cells mixed with islands of hyaline cartilage	+ S100 + SOX9 + Bcl-2 − IDH
Clear Cell	25–50	Epiphyseal, primarily proximal femur and humerus	Well defined, lytic, epiphyseal-based lesions	Sheets of cells with large round nuclei. Cells have a distinct pale or clear cytoplasm	+ S100 + SOX9 + Bcl-2 − IDH

## Data Availability

Not applicable.
